# Is untargeted educational outreach visiting delivered by pharmaceutical advisers effective in primary care? A pragmatic randomized controlled trial

**DOI:** 10.1186/1748-5908-2-23

**Published:** 2007-07-26

**Authors:** Martin P Eccles, Ian N Steen, Paula M Whitty, Lesley Hall

**Affiliations:** 1 Institute of Health and Society, Newcastle University, 21 Claremont Place, Newcastle upon Tyne NE2 4AA, UK

## Abstract

**Background:**

There is increasing evidence that clinical guidelines can lead to improvements in clinical care. However, they are not self-implementing. While educational outreach visits may improve prescribing behaviour, the effectiveness of routine delivery of these visits by existing pharmaceutical advisers is unknown.

**Methods:**

Within a pragmatic randomized controlled trial, involving all general practices in two primary care trusts (PCTs), routine methods were used to distribute guidelines for the choice of antidepressants for the management of depression. Intervention practices were offered two visits (most accepted only one) by their PCT pharmaceutical adviser who had been trained in the techniques of outreach visiting. Intervention practices were visited regardless of whether they had prior problems with prescribing ('untargeted' visits). The intervention was evaluated using level three prescribing analysis and cost (PACT) data for antidepressant drugs for the six months during which the intervention was delivered and the subsequent twelve months.

**Results:**

Across the 72 study practices there was no significant impact of the intervention on usage of any group of antidepressant drugs.

**Conclusion:**

The routine use of untargeted educational outreach visiting delivered by existing pharmaceutical advisers may not be a worthwhile strategy.

**Trial registration:**

ClinicalTrials.gov NCT00393536

## Background

There is increasing evidence that clinical guidelines can lead to improvements in both the process and outcome of care [1]. They figure prominently within the UK, particularly since the inception of the Scottish Intercollegiate Guidelines Network (SIGN) and the National Institute for Health and Clinical Excellence (NICE). However, clinical guidelines are not self-implementing, and there is a growing body of research that demonstrates the effectiveness of various implementation strategies [1]. This has suggested that while the commonly used strategy of the postal distribution of educational materials alone may change clinicians' behaviour, it is unlikely to lead to large changes in practice. Educational outreach visits, using a trained person to meet face-to-face with a health care professional to provide information, may improve practice, especially prescribing behaviour [1,2].

Estimating the effectiveness of educational outreach is complicated by the fact that it is often evaluated as one part of a multi-faceted package, incorporating additional elements such as educational materials, educational meetings, or audit and feedback; Grimshaw *et al*. found this to be the case in all of the 35 studies that they identified in their systematic review [1]. O'Brien *et al *concluded that "educational outreach visits, particularly when combined with social marketing, appear to be a promising approach to modifying health professional behaviour, especially prescribing" [2]. Grimshaw *et al*. found a mixed pattern of results. When considering dichotomous measures of the process of care, they found that educational outreach combined with one other intervention produced a median absolute difference of +2% to +13%. In combination with two other interventions the result for the single study was +11%, and in combination with three other interventions the range of effects was -2% to +6%. The pattern for continuous measures of the process of care was similar, while the effects were consistently smaller for dichotomous measures of outcome of care. There have been 12 trials published since the more recent of these reviews, and their results are consistent with this mixed pattern of effect across settings and targeted conditions [3-14]. However, of the five that focused mainly or exclusively on prescribing, four were negative [6,12-14] and the fifth showed positive effects only in small general practices [11].

Of the five previous studies of the effectiveness of educational outreach visiting in influencing prescribing in the UK NHS [11,12,14-16], three have used pharmacists as the visitors [11,12,14]. This is of particular interest as all primary care trusts (PCTs) in England employ pharmaceutical advisers. They are usually pharmacists whose role is to provide, from a wide clinical and health service management perspective, advice on prescribing and related areas to general practitioners (GPs). Pharmaceutical advisers routinely visit general practices and are seen as change agents; however, the formal delivery of educational outreach by them has not been evaluated.

In 1996, the then Newcastle and North Tyneside Health Authority established a clinical effectiveness unit. The remit of the unit was to provide support to local health care teams in primary and secondary care, with the aim of promoting clinical effectiveness and encouraging the use of best evidence in daily practice through systematic, evidence-based approaches to guideline implementation. The strategy adopted by the clinical effectiveness unit was to concentrate on five clinical areas. These were selected by a multi-disciplinary steering group using explicit criteria (evidence of inappropriate variation in practice; a good evidence base for what should be done; the clinical area should be a source of significant morbidity or mortality; large cost implications in the management of the topic). Depression was one of the clinical areas chosen: depression is an area of significant morbidity (depression affects between 5% and 10% of individuals in the UK and is the third most common reason for consultation in general practice [17]); the costs of antidepressant prescribing had been rising steadily through the 1990s [18].

The aim of this study was to evaluate the effectiveness of outreach visiting by existing pharmaceutical advisers, in addition to the postal distribution of educational materials, for the choice of antidepressants in the management of depression.

## Methods

### Study design

The study was a pragmatic cluster randomized controlled trial based in two PCTs with general practices as the unit of randomization and analysis [19]. Randomization, stratified by PCT, was performed by numbering the practices then allocating them to either intervention or control groups according to a computer-generated random number list. Because the study was restricted to a defined geographical area, the total number of available general practices (73) was pre-determined. (See power calculation for more detail.)

### The guidelines

The aim of the guidelines was to advise GPs on the most cost-effective choice of antidepressants to manage depression in primary care [20]. The guidelines were developed using standard methods by a multi-disciplinary group of GPs, secondary care mental health specialists and pharmaceutical advisers [21]. The key recommendations from the guidelines can be summarised as:

• 'Consider tricyclics first – as they represent a less expensive option, tricyclic antidepressants (TCAs) should be used as the routine first line drug treatment for depression in primary care.'

• The choice of antidepressant should be based on individual patient factors (expanded in the guidelines).

• If the toxic effects of the older TCAs are perceived to be a problem, eg in a patient who has previously taken a drug overdose, then lofepramine is a more cost effective choice than an SSRI.

• The dose of TCAs should be titrated up to the doses suggested (examples given).

• When faced with a patient not responding to first line drug therapy, reasonable options are (five options given).

The guidelines were distributed through the PCT courier or postal system to each individual GP in Newcastle and North Tyneside during the three month period of April to June 1999. This was the only specific intervention that control group practices received.

### Educational outreach visiting

The six pharmaceutical advisers employed by Newcastle PCT and North Tyneside PCT agreed to deliver the outreach visits within the context of the trial. Shortly after the guidelines were distributed, they wrote to all intervention practices with the offer of a visit. This was followed up by a telephone call. The visits took place between July and December 1999. The purpose of the visit was to encourage implementation of the main messages from the guidelines using the principles of outreach visiting [22], in which they received training specifically for the study. Within the visit, they explored GPs' knowledge and patterns of current activity, offered clear behavioural objectives (identifying, investigating, and treating patients), and acknowledged areas of controversy (such as differing treatment regimes and their cost). They used a pre-developed set of educational materials based on the content of the guidelines. These materials concisely represented the issues, although key messages were highlighted and repeated at the end of the session. Two visits were planned, four to six weeks apart. When performing the visits the advisers attempted to see, in each visit, as many of the GPs in a practice as possible.

### Analysis

The analysis was based on routinely available prescribing data (level three prescribing analysis and cost (PACT) data). As the unit of analysis was the practice, this was aggregated to the practice level. Prescribing data for the main categories of antidepressants (see below) from the eighteen months from July 1999 to December 2000 (from the first visits in July 1999 up to and including December 2000, four quarters after the final visits in December 1999) were used for the analysis. Data for the 12 months prior to the intervention were not available. Data for the period during which the intervention was being delivered (July to December 1999) were available, and were included in the model for the primary analysis (see below). PACT data provide total quantity of dose units (tablets or capsules) prescribed, and total costs per practice in each quarter. ASTRO PUs (age, sex and temporary resident originated prescribing units) are designed to weight individual practice populations for age, sex and temporary residents [23]. Two practices may have the same number of patients, but if one has a predominantly older population it will have a higher ASTRO PU value. A practice that had many students registered may have a large list size, but a relatively low ASTRO PU value. ASTROPU values are accepted as a more accurate measure of prescribing need than list size alone, although ASTROPUs are not related to deprivation levels. We used the total number of dose units per practice, per quarter, adjusted for practice size to achieve mean prescribing dose unit per ASTRO PU. We also analysed cost per ASTRO PU. We considered all the main categories of antidepressants as potential indicators, including tricyclic antidepressants (TCAs), selective serotonin reuptake inhibitors (SSRIs), and monoamine oxidase inhibitors (MAOIs). Additionally, we examined lofepramine individually, as this drug was specifically identified within the guidelines as an alternative to TCAs where there were concerns about suicide risk. While it would have been desirable to exclude the use of antidepressants for conditions other than depression (*e.g*., by excluding low doses and/or short courses), this was not possible using PACT data, which provide information only on total quantity and strength.

The analysis, comparing prescribing of antidepressant drugs within those practices offered a visit compared with control practices during the 12 month period following the introduction of the guidelines, was an analysis of covariance, in which the dependent variables were items per ASTRO PU and cost per ASTRO PU.

Drug use was analysed by practice and by quarter. For each practice, we had a series of repeated measures: quarterly data for the six months of the intervention period and the twelve month period following the intervention. Drug use was analysed using multilevel modeling to account for the repeated measures (quarters nested within practices). Variation between practices and variation between quarters within practices were modeled as random effects. In a preliminary analysis, we modeled the trend in drug use across all six quarters. In the main analysis, we modeled the four quarters corresponding to the post-intervention period and included the two quarters data for the intervention period (July through December 1999) as covariates. An indicator variable was defined to take a value of one for observations corresponding to practices randomized to receive the intervention in quarters following the intervention and zero for all other observations. The effect of the intervention was thus modeled as a fixed effect. Interval estimates of effect size are given.

### Power calculation

As reported earlier, the total number of available general practices (73) was pre-determined. With 36 practices randomized to intervention and 37 to control, we determined that we had 80% power to detect an effect size of 0.66 standard deviations in our outcome measures, assuming a type one error rate of 0.05. This equates approximately to a mean difference per ASTRO PU of 0.5 items of lofepramine, 2.4 items of other TCAs, 1.5 items of SSRIs, and 0.05 items of MAOIs.

### Confidentiality issues

The use of aggregated practice level data meant that it was possible to maintain anonymity of individual GPs. The PCTs supplied the prescribing data and, to enable us to identify the intervention and control practices for analysis, codes were developed and used by the PCTs to mark data accordingly. A letter was sent to every GP in the district outlining the study, informing them that anonymous, aggregated PACT data were being used for analysis, and assuring them of confidentiality. A representative from the clinical effectiveness unit and the PCTs each signed this letter. Because patients were not directly involved, and no patient identifiable data were to be included in the final analysis, a formal application to the local ethics committee was, on enquiry, deemed unnecessary.

## Results

All 73 practices in Newcastle and North Tyneside were invited into the study. Of the 73 practices originally randomised (36 intervention and 37 control), one intervention practice opted out prior to receiving a visit, leaving 35 intervention and 37 control practices. The breakdown of number of GP partners per practice for intervention and control groups is provided in Table 1.

**Table 1:** Practice characteristics: number of GP partners in intervention and control practices

	**Number of practices**	
**Number of GP partners per practice**	**Intervention group**	**Control group**

1	5	5
2	5	6
3	10	5
4	4	10
5	4	3
6	3	6
7	3	1
8	1	1
**Total number of practices**	35	37

All intervention practices were visited once. Most practices declined a second visit four to six weeks later, saying that this was too soon after the first visit. Six intervention practices received a second visit within six months of the first one. Pharmaceutical advisers returned records of their visits for 20 of the intervention practices. Visits lasted between 20 and 45 minutes. For ten visits (50%), all partners were present; six visits (33.3%), only one partner was missing, and, for the remainder, two and three partners were missing in one visit each respectively, and four partners were missing for two visits. In some cases, practice managers, community pharmacists and practice nurses also attended.

The level of prescribing of, and costs per ASTRO PU for, TCAs, lofepramine, SSRIs and MAOIs, analysed by quarter and by treatment allocation are shown in Table 2.

**Table 2:** Mean prescribing dose units of TCAs, lofepramine, SSRIs and MAOIs per AstroPU, and costs per AstroPU, per quarter

**Mean number of items prescribed per ASTROPU by quarter by group**													
**Quarter**		**99/00 (2^nd^)**		**99/00 (3^rd^)**		**99/00 (4^th^)**		**00/01(1^st^)**		**00/01 (2^nd^)**		**00/01 (3^rd^)**	

**Drug**	**Group**	**mean**	**sd**	**mean**	**sd**	**mean**	**sd**	**mean**	**sd**	**mean**	**sd**	**mean**	**sd**

**lofepramine**	**Intervention**	0.93	0.86	0.96	0.75	0.93	0.87	0.89	0.77	0.94	0.80	0.93	0.91
	**Control**	1.08	0.74	1.06	0.68	1.02	0.74	1.05	0.67	0.99	0.65	0.98	0.68
	**All**	1.01	0.80.	1.01	0.71	0.98	0.80	0.97	0.72	0.96	0.72	0.95	0.74
**Other TCAs**	**Intervention**	7.33	4.14	7.53	4.16	7.32	4.39	7.16	4.03	7.31	4.23	7.52	4.27
	**Control**	7.70	3.21	7.80	3.12	7.39	2.89	7.58	2.69	7.54	2.58	7.79	2.60
	**All**	7.52	3.67	7.67	3.64	7.35	3.67	7.38	3.39	7.43	3.46	7.66	3.49
**SSRIs**	**Intervention**	6.04	2.32	6.32	2.39	6.44	2.42	6.77	2.41	6.97	2.65	7.36	2.67
	**Control**	6.36	1.75	6.65	2.03	6.74	2.03	7.01	2.17	7.38	2.15	7.86	2.17
	**All**	6.21	2.04	6.49	2.20	6.59	2.22	6.89	2.28	7.18	2.40	7.62	2.42
**MAOIs**	**Intervention**	0.04	0.07	0.05	0.10	0.04	0.06	0.05	0.07	0.05	0.07	0.04	0.07
	**Control**	0.06	0.08	0.06	0.08	0.05	0.06	0.05	0.06	0.04	0.05	0.04	0.05
	**All**	0.05	0.08	0.06	0.09	0.04	0.06	0.05	0.07	0.04	0.06	0.04	0.06
													
**Mean cost items prescribed per ASTROPU by quarter by group**													

**Quarter**		**99/00 (2^nd^)**		**99/00 (3^rd^)**		**99/00 (4^th^)**		**00/01(1^st^)**		**00/01 (2^nd^)**		**00/01 (3^rd^)**	

**Drug**	**Group**	**mean**	**sd**	**mean**	**sd**	**mean**	**sd**	**mean**	**sd**	**mean**	**sd**	**mean**	**Sd**

**lofepramine**	**Intervention**	8.83	6.74	9.21	6.33	9.00	6.98	8.35	6.32	8.45	6.67	8.21	6.48
	**Control**	9.72	6.88	9.64	6.26	9.00	6.86	9.04	6.15	8.65	5.76	8.33	5.87
	**All**	9.29	6.78	9.43	6.25	9.00	6.87	8.70	6.20	8.56	6.18	8.27	6.13
**Other TCAs**	**Intervention**	25.62	15.45	28.76	16.87	28.17	16.37	27.27	15.48	26.47	14.84	26.13	14.40
	**Control**	24.85	7.09	27.84	8.54	26.08	8.18	25.68	7.63	24.20	6.77	24.28	6.88
	**All**	25.22	11.83	28.29	13.18	27.09	12.78	26.45	12.04	25.30	11.40	25.18	11.14
**SSRIs**	**Intervention**	132.60	49.52	132.93	49.09	131.59	45.76	117.65	39.66	109.00	41.21	112.06	42.45
	**Control**	141.25	55.29	142.62	55.80	140.78	54.79	122.39	38.04	114.91	33.15	116.52	38.69
	**All**	137.04	52.38	137.91	52.50	136.31	50.46	120.09	38.63	112.04	37.14	114.35	40.34
**MAOIs**	**Intervention**	0.57	0.97	0.71	1.24	0.55	0.94	0.55	0.88	0.55	0.79	0.51	0.76
	**Control**	0.95	1.17	0.99	1.39	0.78	1.04	0.80	1.11	0.71	0.99	0.68	0.88
	**All**	0.77	1.09	0.85	1.32	0.67	0.99	0.68	1.01	0.63	0.90	0.60	0.82

The results of the main analysis are shown in Table 3. During the period July 1999 to December 2000, across all practices usage of SSRIs rose significantly overall (in intervention and control groups combined; average change per quarter across the six quarters across all 72 practices 0.27; 95% confidence interval (CI) 0.24, 0.30). In contrast, there was a very slight downward trend in MAOIs (average change per quarter -0.004, 95% CI -0.006, -0.002) (Table 3). Also over this period, costs of lofepramine (-0.23, 95% CI -0.34, -0.12), TCAs (-0.28, 95% CI -0.44, -0.12), and MAOIs (-0.08, 95% CI -0.11, -0.04) were reduced.

**Table 3:** Estimates of the effect of the intervention on the prescribing of antidepressants assuming a constant difference between intervention and control practices in the year following the intervention

**Drug**		**Average change per quarter across six quarters and all practices**	**Mean (95% CI) difference between intervention and control practices**
**Tricyclic antidepressants (excluding lofepramine)**	**Items/ASTRO PU**	0.00 (-0.04, 0.04)	0.02 (-0.42, 0.46)
	**Cost (£)/ASTRO PU**	-0.28 (-0.44, -0.12)	1.18 (0.03, 2.33)
**lofepramine**	**Items/ASTRO PU**	-0.01 (-0.03, 0.00)	-0.02 (-0.11, 0.16)
	**Cost (£)/ASTRO PU**	-0.23 (-0.34, -0.12)	0.31 (-0.82, 1.45)
**Selective serotonin re-uptake inhibitors**	**Items/ASTRO PU**	0.27 (0.24, 0.30)	-0.03 (-0.34, 0.27)
	**Cost (£)/ASTRO PU**	-5.92 (-6.89, 4.95)	0.66 (-6.61, 7.94)
**Monoamine oxidase inhinitors**	**Items/ASTRO PU**	-0.004 (-0.006, -0.002)	0.00 (-0.02, 0.02)
	**Cost (£)/ASTRO PU**	-0.08 (-0.11, -0.04)	0.10 (-0.15, 0.35)

The analysis of antidepressant prescribing showed no significant impact of the intervention on usage of TCAs, lofepramine, SSRIs, or MAOIs. Change in prescribing as items per AstroPU for TCAs was +0.02 (95% CI -0.42, 0.46); for lofepramine was +0.02 (95% CI -0.11, 0.16); for SSRIs was -0.03 (95% CI -0.34, 0.27); and for MAOIs was 0.00 (95% CI -0.02, 0.02)(Table 3).

While the costs of TCAs in intervention practices stayed about the same over the period, there was a slight decrease in costs of TCAs in control practices; this difference was significant at the 5% level. The cost of TCAs per AstroPU in intervention practices was estimated to be £1.18 (95%CI £0.03, £2.32) higher than in control practices.

## Discussion

This study showed no effect on the volume of drugs prescribed within a pragmatic evaluation of educational outreach visiting by pharmacy advisers in a service setting. The study is therefore a negative trial, which is at variance with the results of most of the previously reported studies [2]. There are a number of possible explanations for this.

Given that the study was based in a pre-defined geographical area and the number of general practices was therefore fixed, it is possible that the study was underpowered and our negative result was a type two error. This possibility can be considered by examining the different groups of antidepressants separately. For lofepramine, prescribing levels throughout the period of the study were approximately one item per ASTROPU for control practices, and slightly less for intervention practices (Table 2). The estimated effect of the intervention was a change in the prescribing of lofepramine of between -0.11 to +0.15 units (Table 3). The maximum possible change would therefore have been on the order of 10 to 15%. If 15% would have been a clinically important change, then power is a possible explanation for not detecting a significant effect. However, for the period of the study prescribing levels for TCAs were running at around seven to eight items per ASTRO PU across all practices (Table 2). The estimated effect of the intervention was a change of between -0.42 and +0.46 units (Table 3). These 95% confidence limits correspond to changes of 5 to 6% in drug prescribing, so the possibility of a clinically important difference can probably be discounted. Power is therefore unlikely to be an explanation for failing to detect a difference in TCAs.

The outreach visits were delivered in addition to all GPs having received a paper copy of the guidelines, and at the time this study was designed this was felt to be both good practice in disseminating information and an ineffective way of changing behaviour. However, a subsequent review of guideline implementation strategies concluded that there was the possibility of modest effects from the postal distribution of educational materials [1]. Therefore, it is possible that our background intervention could have had a small effect that decreased our power to find an effect from outreach visiting. It is also possible that there was "leaking" of the intervention into control practices by the informal contact of doctors in intervention and control practices. We feel this is unlikely to be a problem for two reasons. Grimshaw *et al*. [1] showed that informal contact between general practices around two clinical areas was low, and any impact of an educational outreach visit would be considerably diluted if it was reported secondhand. Contamination of control practices by contact with the pharmaceutical advisers, who did not use any of the formal materials or methods in control practices, was also considered unlikely.

It is possible that the drug markers may not have been sensitive to underlying change. This is unlikely as such outcome measures have been previously used in positive studies [24]. Additionally, these are data that are routinely fed back to all GPs as part of the PACT data within the standard method of informing their prescribing practice. It is unlikely that the study was not long enough. Depression is a common condition managed in primary care. Even after allowing for cases of mild depression, for whom drug treatment would not be indicated, a full-time GP could expect to see approximately 18 new patients with depression over a 12 month period. Therefore, there should have been several opportunities to decide to prescribe differently. However, while such an incidence rate should of itself be sufficient to find an effect, the fact that this effect becomes much smaller when considered within the overall prescribing for all cases of both incident and prevalent depression means that we may have failed to detect an effect. Since the advent of the quality and outcome framework [25] it would now be feasible to collect data solely on incident cases. Anecdotally, the pharmaceutical advisers reported that during the visits GPs frequently commented that for a proportion of patients they were merely continuing a drug (usually an SSRI) on the advice of secondary care following referral. The proportion of such patients is not known but this would again tend to decrease the effect of an intervention delivered solely in primary care.

It is instructive to compare the intervention we used with those used in previous studies. There are both similarities and differences that may have contributed to our negative result. Very few studies have evaluated educational outreach alone. Although we combined it with the distribution of educational materials, as the control group also received the educational materials, the evaluation was of the additional effect of educational outreach. It is possible (though unlikely) that the positive effects of other studies are due to elements other than educational outreach. For example, educational outreach interventions employed in other studies may not have been as tightly defined as we employed in our study.

Our visitors were pharmacists. Previous studies have suggested that the visitor needs to be credible. One previous study of influencing prescribing behaviour in the UK NHS has found positive effects with pharmacist visitors [11] and two have not [12,14]. However, while lack of credibility is a possible explanation for the lack of effect, we have no way of quantifying this. In most instances, we conducted only a single visit and many practices declined a second visit; previous studies have used a wide range of number of visits from one to weekly for several months. However, there does not seem to be a clear relationship between number of visits and effect, with positive effects coming from studies at either end of the range [2].

Although our intervention adhered to the principles of outreach visiting, the intervention was not targeted at specific barriers to change. Previous studies have used social marketing methods that have allowed clinicians to identify individual barriers to change and their potential solutions [26,27]. Such studies have tended to show larger effects. However, in terms of the content and delivery of the intervention it is hard to disagree with the conclusion of O'Brian *et al*. that "further research is needed ... to identify key characteristics of outreach visits that are important to its success". It is perhaps only with better intervention building that we will be able to understand the active ingredients of behaviour change interventions [28,29].

The coverage of GPs per practice who attended the visits also seems an unlikely explanation for the lack of effect as, for those visits where we have records, either none or few of the GPs were missing.

Our intervention was 'untargeted' in that we did not target those practices or GPs who were in some way problematic prescribers. It is therefore of interest that although our previous study of untargeted outreach visiting also showed no impact [14] another large UK NHS based trial of untargeted outreach visiting showed only small effects [11]. Some previous positive studies have only visited those clinicians whose prescribing was at the high end of the range, and for whom there was the greatest capacity for change [2]. In using untargeted outreach visiting, it is almost certainly the case that a number of visits were received by doctors who were already prescribing optimally.

Finally the context of the study was that of a steady rise in SSRI prescribing and active marketing of these drugs by pharmaceutical companies. Therefore the achievement of the aims of the guidelines used in this study was always likely to be a challenge.

The major strength of this study is that it was a rigorous evaluation of the impact of an intervention delivered using a routine service and evaluated using routinely available data. Such area-wide evaluations have the potential to offer a ready laboratory for the evaluation of a range of behaviour change strategies aimed at healthcare professionals. When they can be conducted using routinely available data, they are also relatively cheap to evaluate. However, there will often be trade-offs to be made between the specificity of routinely available data and its precise relationship to the clinical areas of interest.

The routine use of educational outreach visiting by existing pharmaceutical advisers, untargeted, may not be a worthwhile strategy. Further evaluations could usefully focus on pragmatic evaluations of visits targeted at practices with specific difficulties and consider greater use of social marketing strategies.

## Declaration of competing interests

Martin Eccles is Co-Editor in Chief of Implementation Science. All editorial decisions on this article were made by Co-Editor in Chief Brian Mittman. The other authors declare no competing interests.

## Authors' contributions

The study was conceived by ME. It was designed by ME, LH, and NS. It was run by LH and ME. Pharmaceutical advisers in Newcastle and North Tyneside delivered the intervention. PW obtained the data. NS designed the analysis, to which PW and ME contributed. NS carried out the analysis. PW led the drafting of the paper. All authors commented on successive drafts of the paper. All authors read and approved the final manuscript. ME is the guarantor of the paper.
